# Outcome of Newborns with Tracheoesophageal Fistula: An Experience from a Rapidly Developing Country: Room for Improvement

**DOI:** 10.1155/2022/6558309

**Published:** 2022-12-01

**Authors:** Amal Al-Naimi, Sara G. Hamad, Abdalla Zarroug

**Affiliations:** ^1^Pediatric Pulmonology, Sidra Medicine, Doha, Qatar; ^2^Pediatric Pulmonology, Hamad Medical Corporation, Doha, Qatar; ^3^Surgeon-In-Chief Children's Hospital and Medical Center, Omaha, Nebraska, USA

## Abstract

**Methods:**

This is a retrospective review of the medical electronic charts of patients with TEF that were followed at Sidra Medicine in the state of Qatar. The review included the patients who were operated upon in the period of 2011-2021 but continued to follow at our institution in the period of 2018-2021. Demographic data, associated anomalies, preoperative, operative, and postoperative courses, and growth parameters were collected.

**Results:**

A total of 35 patients with TEF (24 males and 11 females) were collected. 49% were full term. We identified seven patients (20%) with isolated TEF, TEF with VACTERL association in 29% of our patients, other chromosomal anomalies in 17%, or associated with other anomalies (not related to VACTERL) in 34% of the patients. The majority of the patients (94%) were of type C-TEF (TEF with esophageal atresia–EA/TEF). All patients were operated except for one patient who died at 2 days of life due to cardiac complications. Median age at which surgery was performed was 2 days (range 1-270 days). Median follow-up was 32 months (range 7-115 months). Immediate postoperative complications were encountered in eleven patients (33%) and included anastomosis leak in 12%, air leak in 6%, sepsis in 6%, chylothorax in 3%, vocal cord palsy and fistula recurrence (combined) in 3%, and failure of TEF closure in 3% of the patients. Long-term respiratory complications were encountered in 43% of our patients. Long-term gastrointestinal complications included gastroesophageal reflux (GERD) in 63%, dysphagia in 31%, and anastomotic stricture in 34% of the patients. Growth was affected in around a quarter of the patients at 6 months after surgery and 22% at 12-month assessment postoperatively. While only five patients died at our institution, only one was directly related to failure of TEF closure and postoperative complications.

**Conclusion:**

This descriptive study reports the clinical outcome of TEF from a rapidly developing country. The distribution of the patients' characteristics and postoperative complications was almost comparable to those from developed countries. This study would aid in addressing the prognostic factors and establishment of evidence-based management pathways of newborns with TEF to improve the clinical outcome in our center and other pediatric tertiary centers in developing countries.

## 1. Introduction

Tracheoesophageal fistula (TEF) is a rare birth defect. It still represents one of the most common congenital developmental malformations of the digestive tract with a prevalence of 1 in 4,000 births [1]. TEF is caused by incomplete separation of the tracheal bud from the primitive foregut during embryogenesis which occurs between 4th and 5th week of gestation [2]. TEF is classified into five types according to Gross classification [3] which is based on the location of the fistula and its association with esophageal atresia: type A is esophageal atresia (EA) without TEF, type B is esophageal atresia with proximal TEF, type C is esophageal atresia with distal TEF which represents the most common type of TEF, type D is esophageal atresia with proximal and distal TEF, and type E is TEF without esophageal atresia which is the least common type of TEF.

EA/TEF can be suspected prenatally on antenatal ultrasound by absent stomach bubble and polyhydramnios. However, the positive predictive value of the combination of these two signs is only 56%, as reported by Stringer et al. [4]. Symptoms and timing of the presentation depend on the type of TEF. Neonates with esophageal atresia usually present immediately after birth with excessive salivation, choking, and failure of passage of orogastric tube. Whereas, children with H-type present later in life with recurrent chest infections [5]. The presence of additional congenital anomalies is common in patients with TEF and is estimated to be around 50% [6, 7]. These anomalies can occur within the context of genetic syndromes or VACTERL association [8]. The most common associated systematic anomalies are cardiovascular anomalies [9].

The management of TEF is surgical repair which consists of separation of the esophagus from the trachea and closure of the defect in the tracheal wall. The presence of long gap between the atretic segments of the esophagus makes the esophageal repair more complex and challenging [10]. Multiple reports recommended delayed primary repair with staged approach in cases of long gap esophageal atresia or in premature infants with respiratory distress or associated congenital heart disease [11]. Surgical repair can be performed via thoracotomy or thoracoscopically. Thoracoscopic approach requires additional surgical experience, but its advantages include less surgical trauma and faster postoperative recovery [12]. Other procedures such as endoscopic fibrin occlusion, sclerotherapy, and laser coagulation exist; however they were associated with high recurrence rates [13].

Morbidity and mortality rates are dependent on multiple factors related to the patient's condition, associated anomalies, surgical technique, and long-term complications. They are also highly variable between centers. Spitz classification [14] is the most common classification used to predict survival rates and is used as a preoperative predictor to aid in parental counselling and comparison of outcomes among pediatric centers. It is based on the presence of low birth weight <1500 grams and/or major congenital cardiac anomalies. It divided cases into 3 subgroups: group I consists of newborns with birth weight above 1500 grams without a major cardiac anomaly, group II consists of newborns with birth weight below 1500 grams or a major cardiac anomaly, and group III consists of newborns with birth weight below 1500 grams and a major cardiac anomaly. Survival rates of patients with TEF were initially reported by Spitz et al. [14] at 97%, 59%, and 22% in groups I, II, and III, respectively. The survival rates have considerably improved due to advances in anesthesia, surgical, and neonatal intensive care. They currently stand at 98%, 82%, and 50% for group I, group II, and group III, respectively [15].

This study describes our experience, as a developing country, in the management of newborn with TEF. We evaluated the studied cases for the clinical characteristics, postoperative immediate and long-term respiratory and gastrointestinal complications, and mortality rates and compared the results with other regional and international pediatric tertiary centers.

## 2. Methods

We performed a retrospective study and reviewed the medical electronic charts of patients of TEF that were followed at Sidra Medicine, the only pediatric tertiary hospital in the state of Qatar at the time of conduction of the study. Surgical services at our institution were established at the beginning of 2018. The study included the patients who were operated at our institution between 2018 and 2021 in addition to the patients who were operated at Hamad Medical Corporation (the only pediatric surgical center before 2018) prior to that period (2011-2017) and continued to follow at our surgery clinics, as it became the only tertiary surgical center in the state of Qatar.

Medical electronic charts were reviewed for demographic data, associated anomalies, preoperative, operative, and postoperative courses, and growth parameters. Nine patients with deficient files or who lost follow-up at our center were excluded, and these patients went to their home country primary for family relocation.

Statistical analyses were performed using the SPSS (SPSS Inc., Chicago, IL, USA) pocket program. The demographic and clinical characteristics of the patients were compared. Quantitative data were presented as median and range, while qualitative data were demonstrated as frequency and percent (%). The categorical variables were compared with the *t*-test; *P* values <0.05 were considered statistically significant for all analyses.

The study was approved by the Institutional Review Board of Sidra Medicine (IRB#1719230) on the 11^th^ of February 2021.

## 3. Results

A total of 35 patients with TEF were included in our study. It contained 24 males and 11 females (male to female ratio of 2.2 : 1). Around half of the patients (49%) were full terms. Only four patients (11%) were antenatally diagnosed; however, all patients were diagnosed immediately after birth. The type of TEF was classified according to Gross classification into thirty-three patients who had type C, one patient had type A, and one patient had type D. The demographic data and patients' characteristics are summarized in Table 1.

Associated anomalies were identified in most of the patients (80%), with TEF being part of VACTERL association in 29%, while TEF was found in the context of chromosomal or other congenital anomalies in 51% of the patients. The remaining 20% of the patients had an isolated TEF, as shown in Table 2. Cardiovascular anomalies remained the most common (63%) associated systematic anomalies in children with TEF. The distribution of the congenital anomalies by systems is summarized in Table 3.

All patients were operated except for one patient who died at the age of 2 days due to complex congenital heart disease. Twenty-three of patients were operated at our institution in Sidra Medicine. Median age at which surgery was performed was 2 days (range 1-270 days). The majority (89%) of TEF repair was performed through right thoracotomy. Only four patients required staged repair due to long gap EA/TEF, all of which had survived.

Immediate postoperative complications were encountered in eleven of our patients (33%) and were summarized in Table 4.

Clinical outcomes were determined after median follow-up of 32 months (7-115 months). Long-term gastrointestinal complications mainly included gastroesophageal reflux (GERD) in 63% of the patients. Long-term respiratory complications were encountered in 43% of the patients and mainly included tracheomalacia in 17% and recurrent cough in 17% of the patients.

There was no statistical significance between the presence of postoperative complications and gender (*P*value = 1), gestational diabetes (*P*value = 0.80), birth weight below 1500 grams (*P*value = 0.13), chromosomal abnormalities (*P*value = 0.44), or surgical approach (*P*value = 3.65).

Growth was affected in around a quarter of the patients at 6 months after surgery and in 22% at 12-month assessment postoperatively. There was also no statistical significance between the growth retardation at 6 months and gender (*P*value = 0.62), gestational diabetes (*P*value = 0.57), chromosomal abnormalities (*P*value = 0.16), or surgical approach (*P*value = 0.457). However, there was statistical significance between growth retardation at 6 months and birth weight below 1500 grams (*P*value = 0.005).

Encountered long-term complications are summarized in Table 5.

In the thirty surviving patients, the feeding was established orally in nineteen patients (63%), while nine patients were gastrostomy tube fed, and two patients were fed via nasogastric tube.

Spitz classification was used for comparison of our results with other tertiary centers. Number of patients and survival rates are summarized in Table 6.

While five patients died at our institution, only one was related to failure of TEF repair and postoperative complications. Whereas, the cause of death in the other 4 patients was due to the associated cardiac and other congenital anomalies and not related to TEF complications. The median age at death was 60 days (range 2-120 days). The measured mortality rate at our center was 22%. Associated severe malformations, especially cardiac anomalies, were the main causes of death. Nevertheless, after exclusion of those causes, our direct postoperative mortality rate was 4.3% from a leak that caused multiorgan failure.

## 4. Discussion

This review represents the first study in our country and describes the postoperative outcomes. Therefore, it can be considered as the foundation for future prospective studies and the benchmark of our institution in comparison with the international centers.

Although the study was conducted over a relatively short period of time, the distribution of the patients' characteristics and various anatomical types was comparable with other reports. The diagnosis of TEF is usually established in the first 24 hours of life; however, it may be delayed [16]. TEF can even be suspected antenatally with nonspecific findings on prenatal ultrasound [4]. TEF was suspected antenatally in only four patients in our study representing a diagnostic gap that needs to be improved. However, all our patients were diagnosed immediately after birth. Diagnosing these abnormalities early in the fetal stage may allow early recognition and management for these babies.

The management of TEF is surgical ligation of the fistula. The presence of long gap between the atretic segments of the esophagus makes the esophageal repair more complex. Surgical repair can be performed via thoracotomy or thoracoscopy approach. The placement of chest drain postoperatively was performed in all the operated cases despite the consensus against its routine use [17]. Previous studies reported that chest drains are associated with more pain and longer recovery. This can be an area of improvement to be tackled at our institution [17]. However, this is minor in comparison with the other major complications that occurred after TEF repair.

Children with TEF have high risk of gastrointestinal and respiratory complications despite the surgical repair of TEF [18]. These complications are more severe in the first few years of life but may last in varying degrees throughout life [18, 19]. Respiratory outcomes, as a result of functional and anatomical abnormalities, include tracheomalacia/tracheobronchomalacia, bronchiectasis, and recurrent cough [15, 20, 21]. Whereas, gastrointestinal complications include esophageal strictures, motility dysfunction, growth retardation, and gastroesophageal reflux (GERD) [17–19]. These complications were similar to those encountered in our study.

Multiple large retrospective studies looked at outcome of infants with TEF including mortality, esophageal strictures, recurrent fistula, and anastomosis leakage which showed significant variation in perioperative care that may affect the patients' outcomes and mandate standardization of care [19]. Perioperative care of patients with TEF is primarily guided by previous training and personal experience rather than evidence-based practices [22]. The lack of established evidence-based care guidelines contributes to the variation in treatment and likely affects both short and long-term outcomes in TEF patients [23]. We recommend evidence-based guidelines for TEF postoperative care be established.

In other studies, as a comparison, the overall in-hospital mortality was 9% in one of the largest studies, and it revealed lower survival in patients with associated acute respiratory distress syndrome, ventricular septal defect (VSD), birth weight (BW) < 1500 g, time of operation within 24 hours of admission, coexisting renal anomaly, imperforate anus, African American race, and lowest economic status [21].

Our center became the only tertiary hospital over the last 4 years of the conduction of the study. It operated on most of the patients (23 out of 35 over the last 4 years). Therefore, mortality at our center was measured as 22%. This is comparable with the mortality rates in other developing countries in the region [24, 25]; however it is still higher than other international centers in the North America and Western Europe. After exclusion of mortality from severe congenital anomalies, our postoperative mortality rate was 4.3%.

In our study, Spitz classification was used to compare our results with the international results. It was found that the survival rates in our study were comparable with recent studies for groups I and II. Whereas, the comparison of group III was limited as there were only two patients under this category.

Based on our study, the outcome of children with TEF may be improved by enhancing prenatal diagnosis, establishing evidence-based care guidelines, and postoperative care, including postoperative chest drain placement. Additionally, routine and regular follow-up are essential in the early detection and management of TEF complications. More longitudinal data is needed to identify and study any modifiable prognostic factors.

## 5. Conclusion

Tracheoesophageal fistula may cause significant morbidity and mortality. Monitoring the clinical outcome of these patients is essential. This descriptive study shed the light on the clinical outcome of TEF from the only pediatric tertiary center in one rapidly developing country. This would assist in the identification of prognostic factors in our center. This would also help in establishing the standard of care and management in these children to improve their clinical outcome.

## Figures and Tables

**Table 1:** Demographic data.

Demographic	Number of patients (*N*%)
Gender	
(i) Male	24 (69%)
(ii) Female	11 (31%)
Gestational age (GA)	
(i) Full term (>37 weeks GA)	17 (49%)
(ii) Late preterm (32-37 weeks GA)	11 (31%)
(iii) Very preterm (28-32 weeks GA)	6 (17%)
(iv) Extreme preterm (<28 weeks GA)	1 (3%)
Antenatal amniotic fluid	
(i) Normal	17 (49%)
(ii) Polyhydramnios	14 (40%)
(iii) Oligohydramnios	4 (11%)
Antenatal suspicion	
(i) Yes	4 (11%)
(ii) No	31 (89%)
Size for gestational age	
(i) Appropriate for gestational age (AGA)	21 (60%)
(ii) Small for gestational age (SGA)	14 (40%)
Total patients	35 (100%)

**Table 2:** Associated congenital anomalies.

	Number of patients (*N*%)
Chromosomal abnormalities	6/35 (17%)
(i) Trisomy 21	2/35 (6%)
(ii) Trisomy 18	1/35 (3%)
(iii) Klinefelter syndrome	1/35 (3%)
(iv) CHARGE association	1/35 (3%)
(v) Chromosomal deletion	1/35 (3%)
VACTERL association	10/35 (29%)
Other anomalies (not related to above)	12/35 (34%)
(i) Cardiac	10/35 (29%)
(ii) GI (other than TEF)	1/35 (3%)
(iii) GU	1/35 (3%)
Isolated TEF	7/35 (20%)
Total	35/35 (100%)

**Table 3:** Associated congenital anomalies by involved system (some children have multiple anomalies).

	Number of patients (*N*%)
Neurological system	4/35 (11%)
Cardiovascular system	22/35 (63%)
Respiratory system	6/35 (17%)
Gastrointestinal system	5/35 (14%)
Genitourinary system	14/35 (40%)
Musculoskeletal system	2/35 (6%)
Other	1/35 (3%)

**Table 4:** Immediate post-operative complications (one patient who was not operated was excluded).

	Number of patients (*N*%)
Anastomosis leak	4/34 (12%)
Air leak	2/34 (6%)
Sepsis	2/34 (6%)
Chylothorax	1/34 (3%)
Vocal cord palsy and fistula recurrence	1/34 (3%)
Failure of TEF closure	1/34 (3%)
Total	11/34 (33%)

**Table 5:** Long-term complications of TEF (some patients have multiple complications).

Respiratory complications	
	Number of patients (*N*%)
Tracheomalacia	6/35 (17%)
Recurrent chest infections	2/35 (6%)
Bronchiectasis	1/35 (3%)
Recurrent cough	6/35 (17%)
Total	15/35 (43%)
*Gastrointestinal complications*	
GERD	22/35 (63%)
Mild	8/22 (36%)
Moderate	9/22 (41%)
Severe	5/22 (23%)
Dysphagia	11/35 (31%)
Anastomotic stricture	12/35 (34%)
*Growth retardation*	
Growth retardation at time of surgery^∗^	14/34 (41%)
Growth retardation at 6 months after surgery^∗∗^	7/29 (24%)
Growth retardation at 12 months after surgery^∗∗∗^	6/27 (22%)

^∗^Excluding 1 patient who was not operated. ^∗∗^Excluding 5 patients that did not survive, and 1 patient who lost follow-up. ^∗∗∗^Excluding 5 patients that did not survive, 1 patient who lost follow-up, and 2 patients who were below 12 months at time of assessment.

**Table 6:** Survival rate of patients in relation to Spitz risk classification.

Group	Definition	Number of patients	Survival rate
Group I	Birth weight > 1500 g without major cardiac anomaly	26	100%
Group II	Birth weight < 1500 g or major cardiac anomaly	7	81%
Group III	Birth weight < 1500 g and major cardiac anomaly	2	0%

## Data Availability

The clinical data used to support the findings of this study are available from the corresponding author upon request.
